# Segmentation algorithm can be used for detecting hepatic fibrosis in SD rat

**DOI:** 10.1186/s42826-023-00167-2

**Published:** 2023-06-28

**Authors:** Ji-Hee Hwang, Minyoung Lim, Gyeongjin Han, Heejin Park, Yong-Bum Kim, Jinseok Park, Sang-Yeop Jun, Jaeku Lee, Jae-Woo Cho

**Affiliations:** 1 Toxicologic Pathology Research Group, Department of Advanced Toxicology Research, Korea Institute of Toxicology, Daejeon, 34114 Korea; 2 Department of Advanced Toxicology Research, Korea Institute of Toxicology, Daejeon, 34114 Korea; 3 Research and Development Team, LAC Inc., 07807 Seoul, Korea

**Keywords:** Digital pathology, Artificial intelligence, Liver fibrosis, Cirrhosis, NDMA, NASH, Image segmentation

## Abstract

**Background:**

Liver fibrosis is an early stage of liver cirrhosis. As a reversible lesion before cirrhosis, liver failure, and liver cancer, it has been a target for drug discovery. Many antifibrotic candidates have shown promising results in experimental animal models; however, due to adverse clinical reactions, most antifibrotic agents are still preclinical. Therefore, rodent models have been used to examine the histopathological differences between the control and treatment groups to evaluate the efficacy of anti-fibrotic agents in non-clinical research. In addition, with improvements in digital image analysis incorporating artificial intelligence (AI), a few researchers have developed an automated quantification of fibrosis. However, the performance of multiple deep learning algorithms for the optimal quantification of hepatic fibrosis has not been evaluated. Here, we investigated three different localization algorithms, mask R-CNN, DeepLabV3^+^, and SSD, to detect hepatic fibrosis.

**Results:**

5750 images with 7503 annotations were trained using the three algorithms, and the model performance was evaluated in large-scale images and compared to the training images. The results showed that the precision values were comparable among the algorithms. However, there was a gap in the recall, leading to a difference in model accuracy. The mask R-CNN outperformed the recall value (0.93) and showed the closest prediction results to the annotation for detecting hepatic fibrosis among the algorithms. DeepLabV3^+^ also showed good performance; however, it had limitations in the misprediction of hepatic fibrosis as inflammatory cells and connective tissue. The trained SSD showed the lowest performance and was limited in predicting hepatic fibrosis compared to the other algorithms because of its low recall value (0.75).

**Conclusions:**

We suggest it would be a more useful tool to apply segmentation algorithms in implementing AI algorithms to predict hepatic fibrosis in non-clinical studies.

## Introduction

Liver fibrosis is an abnormal repair reaction in chronic liver injury characterized by the excessive production and accumulation of extracellular matrix (ECM) in the liver. It is caused by chronic hepatitis B (CHB), chronic hepatitis C (CHC), alcoholic fatty liver disease (AFLD), and other causes [1–4]. Liver fibrosis begins with pro-inflammatory reactions; liver tissues’ standard structure and physiological function are gradually destroyed. This causes the production of scar tissue that replaces the liver parenchyma and further progress into more severe consequences, such as liver cirrhosis, liver failure, or liver cancer, eventually leading to patient death of patients [5]. However, liver fibrosis is reversible in the early stages of cirrhosis; therefore, it is a top priority in treating liver conditions. This treatment aims to reduce or reverse hepatic fibrosis by reducing inflammation, protecting the liver, preventing the proliferation and activation of hepatic stellate cells (HSCs), and restraining ECM production and deposition [6, 7]. Many antifibrotic candidate drugs have shown reliable results in experimental animal models; however, they have shown limited effects in the clinical phase owing to the complicated pathological mechanisms of liver fibrosis. Adverse reactions induced by large doses are one of the leading causes of failure. Most drugs targeting liver fibrosis caused by various factors in chronic liver diseases are still in the preclinical stage of development [8].

Rodent models have usually been used to evaluate the efficacy of anti-fibrotic therapeutics in non-clinical research in which histopathological differences between the control and treatment groups are examined. The accurate quantification of liver fibrosis is pivotal for assessing the efficacy of novel anti-fibrotic candidates. Conventionally, semi-quantitative histological evaluation has been the method of choice for liver fibrosis assessment [9, 10] and is still regarded as the gold standard. In the last two decades, there have been significant progress in digital image analysis (DIA) for analyzing biopsy specimens. Researchers have focused on developing automated methods to quantify fibrosis by determining the ratio of fibrosis areas to the total area of liver tissue examined. This is done using a measurement called the proportionate collagen area (CPA), which calculates the extent of fibrosis in relation to the entire liver tissue area analyzed. [11–14]. Furthermore, recent studies have started to adopt deep learning methods to score hepatic fibrosis in rodent models [15–17]. These methods have shown reliable correlations with pathologist scoring systems, even at the whole-slide image (WSI) level [17]. However, it is crucial to evaluate the effectiveness of different artificial intelligence (AI) algorithms and determine the most appropriate AI before implementing a specific algorithm for pathological use.

As previous studies have shown, localizing and separating the lesion of interest on the slide is essential to quantify and visualize abnormalities [17, 18]. However, those studies used only one algorithm, not the other detection type of algorithm. Therefore, in this study, we examined the performance of three different localization algorithms for detecting hepatic fibrosis: SSD [19], an object-detection algorithm, and two segmentation algorithms: Mask R-CNN [20] and DeepLabV3^+^ [21]. We considered Mask R-CNN and DeepLabV3^+^ because of the morphology of fibrosis, atypical and polygonal shape; thus, we assumed that the segmentation algorithm would be more efficient in recognizing the lesion. In contrast, SSD was selected because of its fast speed in detecting an objective in an image in real-time. Pathologists can diagnose a slide quickly by using a microscope to determine whether the slide has fibrotic lesions. Therefore, if the SSD shows an accuracy comparable to that of the segmentation algorithms, it could be more valuable than the others. In this study, to investigate the proper deep learning algorithm for detecting liver fibrosis, we evaluated the performance of each model using precision and recall based on predicting fibrosis on large-scale images rather than trained images.

## Results

### Algorithm training

**Fig. 1:**
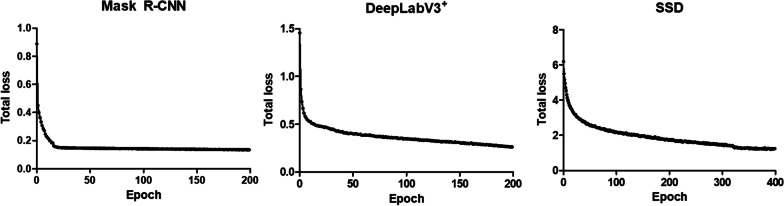
Total loss according to algorithm models observed in every epoch during the training

All the losses during training were calculated and recorded as total losses. Although the loss components calculated during training differed according to the algorithm, the loss values stabilized steeply during the early phase of learning (Fig. 1). The loss values observed in this study show that algorithm learning was successfully performed using the training dataset. After the model training, each algorithm’s mean intersection of union (mIoU) was calculated for the test dataset. Consequently, the mIoU value of the two segmentation algorithms was 0.76, comparable to the ground-truth annotations, and that of the SSD was 0.82.

### Model accuracy

**Fig. 2:**
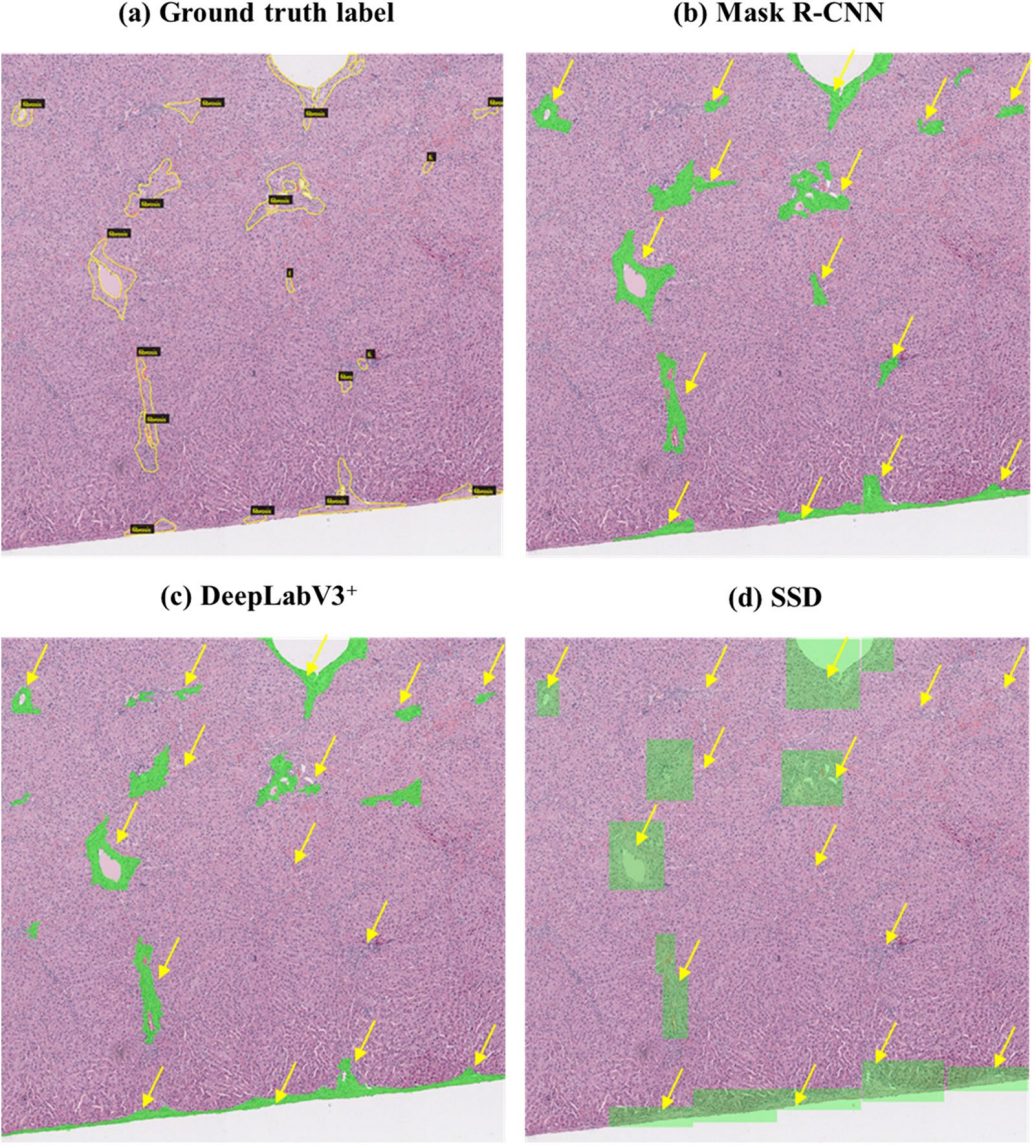
Prediction result of each trained algorithm on 2688*2688 pixels of images. Yellow arrows point to the region of ground truth labels. Mask R-CNN detected hepatic fibrosis the most similar to the ground truth labels

According to the trained weight (Fig. 1), the results showed that the two segmentation algorithms (Mask R-CNN and DeepLabV3^+^) predicted hepatic fibrosis closer to the ground truth label than the object detection algorithm SSD; in particular, the trained Mask R-CNN algorithm showed the closest prediction to the ground truth annotation compared with other algorithms (Fig. 2).

We also calculated the precision, recall, F1 score, and accuracy based on the ground-truth labels to mathematically evaluate the model’s performance.

The results showed that the performances of the two segmentation models were better than that of the SSD object detection model. Similar values were obtained to evaluate the prediction accuracy of each algorithm. However, the algorithms had differences in recall values (Table 1). The Mask R-CNN showed the highest values for all parameters related to the model performance for detecting hepatic fibrosis and the highest recall value. The performance of the SSD model was lower than that of the segmentation algorithms showing the lowest recall value when compared with the two segmentation algorithms. This indicates that SSD is limited in detecting hepatic fibrosis close to the ground-truth annotation.

**Table 1:** Precision, recall, F1 score, and accuracy were calculated from a large-scale image prediction test

	Precision	Recall	F1 score	Accuracy
Mask R-CNN	0.82	0.93	0.87	0.86
DeepLabV3^+^	0.81	0.88	0.84	0.83
SSD	0.83	0.75	0.79	0.79

## Discussion

To investigate the proper deep learning algorithm for detecting hepatic fibrosis in a non-clinical study, we investigated three different localization algorithms for detecting hepatic fibrosis: SSD, mask R-CNN, and DeepLabV3^+^. A total of 5750 images with 7503 annotations were trained using the three algorithms, and the total loss in each model was observed during training. Loss occurs because of the estimation error of the model when a learned model is applied to real data; therefore, the smaller the loss, the better the model. The two segmentation algorithms, Mask R-CNN and DeepLabV3^+^, showed smaller loss value ranges than the object detection algorithm SSD; thus, the training of the two former algorithms was more successful than that of SSD. After training, the mean interaction of union (mIoU) of the test dataset was calculated by comparing the annotations to the prediction results that refer to the trained weight of each algorithm. There was a difference in the calculation method for the intersection of union (IoU) between the two segmentation algorithms and SSD. A segmentation algorithm compares the annotation to the prediction results based on the area, whereas the SSD uses the prediction rate for the number of labels. Therefore, the IoU values of the segmentation algorithms can vary according to the IoU between the prediction by the trained model and the ground truth label. In the case of SSD, the IoU was defined by three values (1, 0.5, and 0.33) according to the prediction rates of 100%, 50%, and 33% of the predicted hepatic fibrosis, respectively, compared with the number of ground truth labels. Therefore, it tends to be overestimated compared with the IoU values from the segmentation algorithms, which are calculated based on the area and region between the ground truth and the prediction. Thus, the mIoU calculated from the three algorithms had a limitation in comparing their performances.

To overcome this limitation and confirm its performance on a large-scale image, we evaluated the precision, recall, F1 score, and accuracy of the predictions of 2688 × 2688 pixel images for each trained algorithm. The trained Mask R-CNN outperformed the other algorithms in predicting hepatic fibrosis, although it mispredicted inflammatory cells and connective tissue as hepatic fibrosis (Fig. 2b). The trained DeepLabV3^+^ tended to detect inflammatory cells and connective tissue in hepatic fibrosis better than the trained Mask R-CNN (Fig. 2c). The trained SSD showed the lowest performance in detecting hepatic fibrosis among the two segmentation algorithms. Blood vessels were not excluded from the images (Fig. 2d).

The parameters related to the model accuracy also proved the segmentation algorithms’ high performance compared to the objective detection algorithm. A high recall value indicated that detecting hepatic fibrosis by the trained algorithm was closest to the ground truth. The trained Mask R-CNN exhibited good performance on the test images. This tendency is reflected well in the prediction results in Fig. 2, where the Mask R-CNN identified the inflammatory lesions and connective tissue from hepatic fibrosis better than the other segmentation algorithm, DeepLabV3^+^. Therefore, the trained Mask R-CNN results for detecting hepatic fibrosis in the test image were the closest to the ground truth label and showed the highest accuracy compared to any other model. The other segmentation algorithm, DeepLabV3^+^, used in this study, DeepLabV3+, performed comparably to the Mask R-CNN. However, it has a recall limitation owing to the frequent misprediction of inflammatory cells and connective tissue in hepatic fibrosis.

In contrast, the trained SSD, an object detection model, showed the lowest values related to model accuracy compared with the segmentation algorithms, especially regarding the recall and the ability to predict hepatic fibrosis compared with ground truth annotations. This result is presented in Fig. 2d as empty detection results with yellow arrows, indicating that the trained SSD did not predict hepatic fibrosis as well as the other algorithms. Indeed, the bounding-box-based detection algorithm might be suitable for detecting an object that can be filled in the bounding box, such as an automobile, but not for atypical and long-shaped objects, such as hepatic fibrosis. Therefore, the SSD may not be a suitable algorithm for detecting hepatic fibrosis.

A previous study by Ramot et al. [16] demonstrated automated quantification of liver fibrosis in mice using a segmentation algorithm, U-net, with two magnifications (10× and 40×) of picrosirius red-stained slide images. The F1 score of the study (0.8775) was similar to the value observed in this study (0.87 for Mask R-CNN), although the staining method and trained algorithm were different [16]. This result supports our previous study [17], which showed the possibility of applying a Mask R-CNN to quantify hepatic fibrosis at the WSI level. In addition, the results from this study showed again that the implementation of Mask R-CNN could successfully quantify hepatic fibrosis using H&E staining, a general staining method for tissue analysis, instead of specific staining, such as Sirius red or Masson’s trichrome staining.

## Conclusions

In this study, the Mask R-CNN outperformed the others in detecting hepatic fibrosis, especially regarding the recall value; therefore, it showed the closest prediction results among the algorithms. The other segmentation algorithm, DeepLabV3^+^, showed comparable accuracy to the Mask R-CNN; however, it showed a lower prediction rate for detecting hepatic fibrosis than the Mask R-CNN. SSD showed the lowest accuracy and ability to predict hepatic fibrosis compared with the segmentation algorithms. Therefore, we suggest that segmentation algorithms can help to implement artificial intelligence algorithms to predict toxicological lesions in non-clinical studies.

## Methods

### Animal experiments

N-nitrosodimethylamine (NDMA) was administered to the test animals via a four-week repeated intraperitoneal injection to induce hepatic fibrosis in Sprague-Dawley (SD) rats. Details of the animal experiments have been described previously [17]. Briefly, 1 mL/kg NDMA (10 mg/10 mL) was administered to 6-to 7-week-old SD rats via intraperitoneal (IP) injection three times a week for four weeks (total of 12 times). After chemical administration, the test animals were euthanized using isoflurane, and their livers were collected in 10% formaldehyde. After tissue collection, hematoxylin and eosin (H&E) staining was performed using paraffin-embedded left lateral and median lobes of the liver, and the sections were used for digital archiving.

### Data preparation

Data preparation for training on hepatic fibrosis was conducted as described in previous studies [17, 18]. Briefly, 10× magnified whole-slide images (WSIs) of liver sections were cropped into 448 × 448 pixel tile images, and all lesions were labelled. We annotated all fibrotic lesions in the tile images using VGG Image Annotator 2.0.1.0 (Visual Geometry Group, Oxford University, UK), and an accredited toxicological pathologist confirmed the annotations before algorithm training was initiated. The annotation information was saved in a JSON file. A total of 500 image tiles were obtained from 12 WSIs. The lesions identified in these images were labeled, and 663 annotations were obtained. The training test split function embedded in the scikit-learn package was used to split the annotated image tiles into training, validation, and test datasets at a ratio of 7:2:1. Data augmentation was conducted to improve the training dataset. It was performed 16 times using image-augmenting techniques (reverse, rotation, and brightness). A total of 5600, 100, and 50 images were used for training, validation, and testing, respectively, and the number of annotations was 7296, 140, and 67, respectively.

### Training of hepatic fibrosis and metrics for model performance

#### Model training

TensorFlow 2.1.0, Keras 2.4.3 backend, and PyTorch were used for conducting algorithm training. We applied three open-source packages (Mask R-CNN: torchvision [22], DeepLabV3+: jfzhang95 pytorch-deeplab-xception package [23], SSD: amdegroot ssd.pytorch package [24]) to train the hepatic fibrosis, and all the requirements for the packages were met in this study. Algorithm calculation during the training was powered by an NVIDIA RTX 3090 24G GPU. The hyperparameters were set differently for each algorithm because of the varying hyperparameter requirements according to the algorithm, and Mask R-CNN used the same settings as in previous studies [17, 18]. Details are presented in Table 2. Every loss calculated using the algorithm during training was recorded and saved.

**Table 2:** Hyperparameters used in Mask R-CNN, DeepLabV3^+^, and SSD

Mask R-CNN	DeepLabV3^+^	SSD
BACKBONE = resnet152	BACKBONE = “resnet”	COCO_API = “PhythosAPI”
DIST_BACKEND = “nccl”	LR = 0.01	EPOCHS = 400
BATCH_SIZE = 16	EPOCHS = 200	BATCH_SIZE = 64
LR = 0.005	BATCH_SIZE = 32	LR = 0.00003
EPOCHS = 200	DATASET = “coco”	WEIGHT_DECAY = 0.00003
WORKERS = 16	MASK_THRESHOLD = 0.6	MOMENTUM = 0.9
LR_STEPS = 16, 22		CONFIDENCE = 0.5
CONFIDENCE = 0.5		

#### Metrics for model performance

**Fig. 3:**
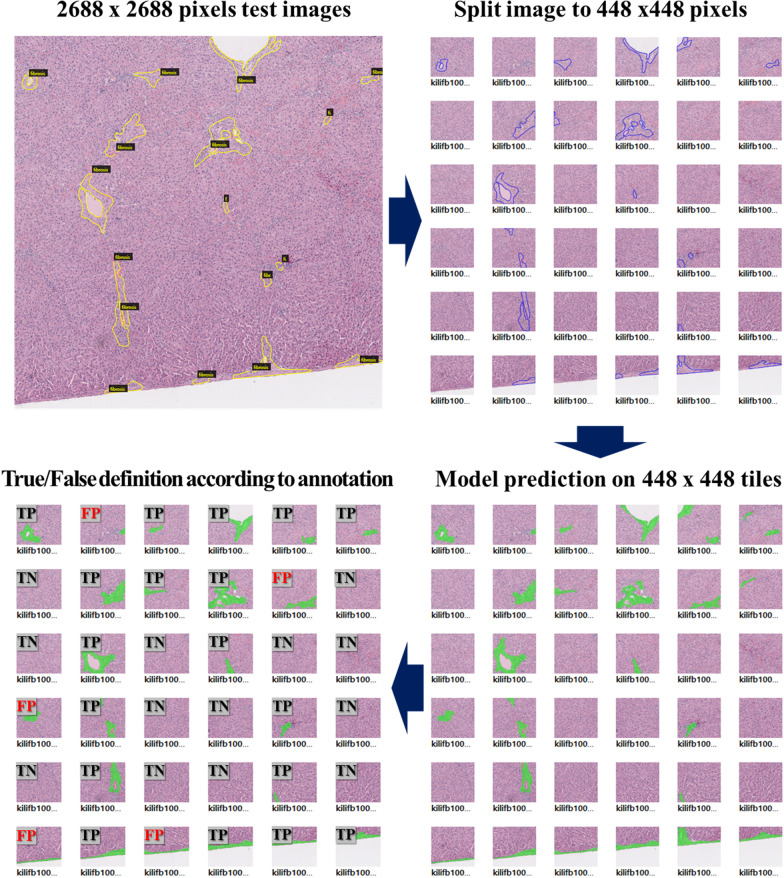
The process to calculate the parameters regarding the examination of model performance in large-scale images. True and false are determined by comparing the ground truth annotation to the prediction results according to the weight of each model at the level of 448 × 448 pixels of tiles

To evaluate the performance of each trained model, we compared the precision, recall, F1 score, and accuracy calculated from the prediction of hepatic fibrosis using 60 large-scale images (2688 × 2688 pixels). First, the ground truths of the test images were annotated using the same procedure used to prepare the training data to calculate these values. Then, the values were defined as the ratio of true positives, false positives, and false negatives according to the presence or absence of lesion detection in the 448 × 448 pixels of tiles derived from 2688 × 2688 images compared to the ground truth labels. A schematic diagram for calculating the precision, recall, F1 score, and accuracy in larger-scale test images is depicted in Fig. 3. The precision, recall, and accuracy are defined as follows (a–d):

- $$\text{Precision}=\frac{TP}{TP+FP}$$
    
- $$\text{Recall}=\frac{TP}{TP+FN}$$
    
- $$\text{Accuracy}=\frac{TP+TN}{TP+FN+FP+TN}$$
    
- $$\text{F1 score}=\frac{2*Precision*Recall}{Precision+Recall}$$

## Data Availability

The datasets generated and(or) analyzed during the current study are not publicly available because they are currently under copyright registration but are available from the corresponding author upon reasonable request.

## Abbreviations

AFLD: Alcoholic fatty liver disease
CHB: Chronic hepatitis B
CHC: Chronic hepatitis C
CPA: Collagen proportionate area
DIA: Digital image analysis
ECM: Extracellular matrix
H&E: Hematoxylin and eosin
HSC: Hepatic stellate cells
IoU: Intersection of union
mIoU: Mean intersection of union
NDMA: N-nitrosodimethylamine
WSI: Whole slide level
